# Major Etiological Agents Isolated from Neonatal Calf Diarrhea Outbreaks in Northern Italy

**DOI:** 10.3390/pathogens14090847

**Published:** 2025-08-25

**Authors:** Camilla Torreggiani, Giovanni Pupillo, Chiara Anna Garbarino, Gianluca Rugna, Alice Prosperi, Chiara Chiapponi, Andrea Luppi

**Affiliations:** Experimental Zooprophylactic Institute of Lombardy and Emilia-Romagna (IZSLER), “Bruno Ubertini”, 25124 Brescia, Italy

**Keywords:** bovine coronavirus, bovine rotavirus, *Cryptosporidium parvum*, enterotoxigenic *E. coli*, neonatal calf diarrhea

## Abstract

Neonatal calf diarrhea (NCD) represents a major cause of economic loss in dairy cattle herds worldwide. The condition is primarily associated with several key pathogens, including enterotoxigenic *Escherichia coli* (ETEC), viral agents such as bovine rotavirus (BRV) and bovine coronavirus (BCoV), and the protozoan *Cryptosporidium parvum*. This study aimed to assess the prevalence of NCD-associated pathogens in Italian dairy farms over the period 2020–2022. Among the 598 farms affected by NCD and included in the investigation, ETEC strains were detected in 17.2% of cases. The prevalence of BRV, BCoV, and *Cryptosporidium* spp. was 22.2%, 20.2%, and 32.3%, respectively. Co-infections were also frequently observed and are considered to significantly exacerbate the clinical severity of the disease. Ongoing surveillance of NCD pathogens is essential to generate reliable and updated epidemiological data, which are critical for guiding effective control and prevention strategies.

## 1. Introduction

Neonatal calf diarrhea (NCD) represents the principal cause of mortality in neonatal calves, accounting for over 50% of total calf deaths [1,2,3,4]. Beyond its impact on survival, NCD contributes substantially to economic losses through reduced weight gain, increased treatment costs, and the need for preventive measures such as antimicrobial therapy and vaccination. The syndrome is multifactorial in origin, involving complex interactions among infectious agents—including viruses, bacteria, and protozoa—and management-related factors such as housing, nutrition, and hygiene [5]. The four most common diarrhea-causing agents in neonatal calves are enterotoxigenic *Escherichia coli* (ETEC)*,* viral pathogens such as bovine rotavirus (BRV) and bovine coronavirus (BCoV), and *Cryptosporidium parvum* [6,7,8,9].

Enterotoxigenic *E. coli* is a primary contributor to intestinal disease in calves, especially within the first four days of life [1,10,11,12]. ETEC encode lipopolysaccharide structures that may act as endotoxins, fimbrial adhesins, and finally enterotoxins. Fimbrial adhesins F5, F17, and F41 are linked to diarrhea in calves [11]. Regarding enterotoxins, although the detection of LT toxins is common in ETEC strains affecting other species, such as swine, they are almost absent in ETEC strains associated with calf diarrhea [11]. In calves, only the heat-stable toxin (ST) is implicated in the pathogenesis of diarrhea. The endotoxins in the bloodstream induce fever, harm endothelial cells, and trigger disseminated intravascular coagulation, which results in acute shock and sudden death [1,13]. The target sites of fimbriae on the immature enterocytes decrease from 12 h onwards [14]. The relatively short incubation time of 12 h and the possibility to adhere to the immature enterocytes make it the most prevalent underlying pathogen of NCD in calves less than five days old [15].

Regarding viral agents, bovine rotavirus (BRV) and bovine coronavirus (BCoV) are the primary viruses implicated in neonatal calf diarrhea. BRV typically affects calves between 4 and 14 days of age. The virus invades and damages villous epithelial cells in the small intestine, leading to nutrient malabsorption [15]. In addition, BRV produces the enterotoxin NSP4, which alters fluid and nutrient transport across the intestinal epithelium, resulting in hypersecretory diarrhea [16]. Virus excretion by cows is particularly common around the time of calving, facilitating the persistence of infection within herds [1]. BCoV-associated diarrhea generally occurs in calves aged 4 to 30 days and, although less frequent than BRV infection, can cause severe disease [16]. The virus induces enterocolitis characterized by villous enterocyte destruction in the small intestine and damage to ridges and crypts in the large intestine. The resulting maldigestion, malabsorption, and inflammation contribute to the pathophysiology of BCoV diarrhea [16]. Regarding *Cryptosporidium parvum*, calves are most susceptible during their second week of life, and infections acquired earlier are associated with a longer patent period [8,17]. This protozoan primarily affects nursing calves younger than three weeks and is strongly associated with NCD [18,19]. *C. parvum* is transmitted via the fecal–oral route, either through direct contact with feces from infected animals or indirectly via contaminated environments, feed, or water [20]. Infections and associated gross lesions are most frequently observed in the small intestine, but may also be present in the duodenum, caecum, and colon. Villous atrophy, crypt hyperplasia, and epithelial cell damage contribute to the malabsorptive diarrhea characteristic of the infection. The high environmental resistance of oocysts is a key factor in the epidemiology of this protozoan, facilitating its long-term survival and continued transmission [17,20].

Mixed infections involving multiple enteric pathogens play a critical role in the pathogenesis of neonatal calf diarrhea (NCD), often exacerbating disease severity and complicating therapeutic management. While individual agents such as rotavirus, coronavirus, *Escherichia coli*, and *Cryptosporidium parvum* are well-recognized causes of NCD, their concurrent occurrence has been associated with more pronounced clinical signs, prolonged diarrheal episodes, and higher mortality rates [6,15,18]. Evaluating mixed infections is therefore essential, as their synergistic effects may influence both the course of disease and the effectiveness of control measures, ultimately impacting animal welfare and farm productivity.

As previously mentioned, NCD is influenced by a complex interplay of enteropathogens and environmental factors. Understanding the specific contribution of each factor is crucial for the development of targeted diagnostic approaches and the implementation of effective control measures, underlining the importance of a thorough etiological diagnosis. Although not specifically addressed in this study, pathogens such as bovine norovirus and nebovirus may also play a contributory role in the pathogenesis of neonatal calf diarrhea (NCD). Several European studies have reported relatively high prevalence rates for bovine norovirus, including 11% in the U.K. [21] and 20% in Sweden [22], suggesting that their epidemiological and clinical significance should not be underestimated and warrant further investigation in future studies. To the best of the authors’ knowledge, no previous studies have investigated the prevalence of neonatal diarrhea pathogens in calves within the specific geographical area covered by this study; therefore, the objectives of this study were (i) to assess the prevalence of four major NCD pathogens in calves aged 1–10 days from dairy farms in the Emilia-Romagna region of Italy, an area of strategic importance as the second-largest Italian region for dairy cattle population, between 2020 and 2022 and (ii) to provide updated insights into the most common ETEC virotypes circulating in diarrheic calves in this geographic area.

## 2. Materials and Methods

### 2.1. Description of the Calf Rearing Area

**Figure 1:**
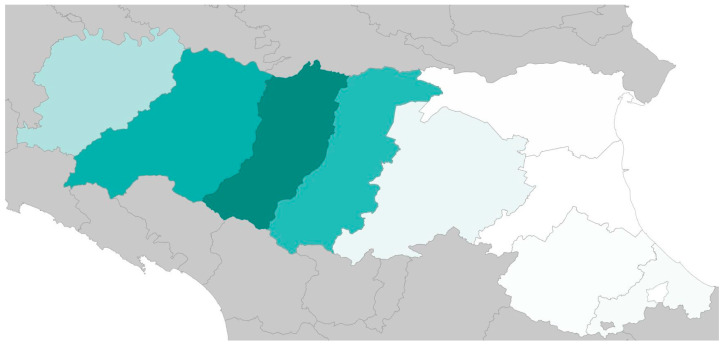
The figure illustrates the density of dairy farms in the Emilia-Romagna region, indicating an even distribution of farms across the area. Areas with a greater concentration of dairy farms are represented by darker colors. This map is sourced from the national database of the Italian Ministry of Health [23].

The Emilia-Romagna region, situated in the Po Valley of northern Italy, is characterized by predominantly flat topography and a dense, well-developed agricultural infrastructure. It represents one of the most intensive dairy farming areas in the country and holds the second-largest dairy cattle population in Italy (Figure 1). Dairy production in this region plays a central role in the regional economy, particularly due to its association with the production of Parmigiano-Reggiano and Grana Padano cheese.

The farms included in the present study are highly specialized and exclusively dedicated to milk production for Parmigiano-Reggiano and Grana Padano manufacturing, adhering to strict feeding and management protocols required by the Protected Designation of Origin (PDO) guidelines. The majority of calves enrolled were purebred Italian Friesian Holsteins, a breed selected for high productivity and adaptability to intensive farming systems. A minority of crossbred animals was also represented.

Outbreaks of neonatal calf diarrhea were reported throughout the year, highlighting the persistent nature of enteric infections in this intensive dairy context and the ongoing challenges posed to calf health management strategies in the region.

### 2.2. Detection of Etiological Agents Isolated from Neonatal Calf Diarrhea

Between 2020 and 2022, a total of 672 preweaned calves, all under 10 days of age and originating from 598 dairy herds in Italy, were submitted to the diagnostic laboratories of the Experimental Zooprophylactic Institute of Lombardy and Emilia-Romagna (IZSLER) in the Emilia-Romagna region. These cases were part of routine postmortem investigations aimed at determining the cause of mortality following the onset of diarrhea. Postmortem examination of the calves included in this study revealed that over 70% presented marked dehydration and extensive fecal soiling of the perineal region, tail, and hind limbs. Additionally, the body condition score was uniformly low, reflecting a poor nutritional status. A comprehensive overview of the principal gross pathological findings observed during necropsy in all examined calves is detailed in Table 1. Calves younger than 10 days of age were included in order to specifically target the period of highest incidence of NCD and greatest susceptibility to infection. Restricting the age range also minimized variability related to immune maturation and the shifting prevalence of pathogens in older calves. Although most studies include animals up to 21–30 days of age, our approach was deliberately designed to provide a more focused analysis of the earliest and most critical phase of the disease. The selection of this specific population allowed for a targeted evaluation of the prevalence and impact of four major pathogens commonly associated with neonatal calf diarrhea, particularly considering the limited pathogenic impact of enterotoxigenic *Escherichia coli* in older calves. During necropsy, fecal samples and intestinal tissues were collected from diarrheic calves and processed according to standardized diagnostic protocols. For the detection of *E. coli*, specimens were cultured on blood agar and Gassner agar, a differential medium used to distinguish lactose-fermenting bacteria, such as *E. coli*, from non-lactose-fermenting Gram-negative bacteria. Plates were incubated aerobically at 37 °C for 24 to 48 h [24]. Colonies exhibiting morphological and staining characteristics consistent with *E. coli* were subsequently confirmed using a miniaturized biochemical identification system (EnteroPluri-Test, Liofilchem S.r.l., Italy). Confirmed isolates underwent molecular characterization by multiplex polymerase chain reaction (PCR) targeting key virulence-associated genes of presumed importance in cattle, including those encoding fimbrial adhesins (F4/K88, F5/K99, F6/987P, F18, F41) and enterotoxins (LT, STa, STb) [25]. Isolates harboring at least one fimbrial and one toxin gene were classified as ETEC. To avoid data inflation, only one representative strain per virotype was included for each outbreak. For viral agents, real-time PCR assays were performed on fecal samples to detect BRV [26] and BCoV [27] (Table 2). Samples were homogenized 1/10 *v*/*v* in phosphate-buffered solution (PBS), and viral RNA was extracted from 100 µL of homogenized samples using a One for All vet kit (Indical Bioscience GmbH, Leipzig, Germany), followed by the addition of 5 µL of Internal Control RNA (High concentration) (QIAGEN, Milan, Italy) according to manufacturer’s instructions. Multiplex real-time RT-PCR for BRV A and BCoV was performed using a one-step reaction with primers and probes targeting conserved genomic regions. The 25 µL reaction included 5 µL of RNA template, 0.2 µM of each primer and probe (Table 2): Primer BRV-F5, Primer BRV-F6, BRV-Probe, BCoV-fwd, BCoV-rev, BCoV-Probe, 1x of 5X QuantiFast^®^ Pathogen Master Mix (QIAGEN, Milan, Italy), 1x of QuantiFast^®^ Pathogen RT Mix (QIAGEN, Milan, Italy), and 1x of Internal Control Assay (QIAGEN, Milan, Italy). The cycling conditions were as follows: a reverse-transcription step of 20 min at 50 °C, followed by denaturation at 95 °C for 5 min, and then 45 cycles of 94 °C for 15 s and 60 °C for 30 s for elongation/fluorescence detection.

Samples were considered positive when the cycle threshold (Ct) value ranged between 5 and 38. The detection of *Cryptosporidium* spp. was carried out using the modified Ziehl–Neelsen staining method, enabling microscopic identification of oocysts [28]. Our testing method does not allow for the specific identification of the Cryptosporidium species detected; consequently, the results will be reported as *Cryptosporidium* spp.

## 3. Results

The calves included in our study were sampled at an early age to capture the critical period of susceptibility to enteric infections. The mean age of the sampled calves was 7.3 days, ranging from 1 to 10 days. Notably, all samples tested positive for at least one of the four targeted enteropathogens, highlighting the high prevalence of these pathogens in neonatal calves.

A total of 498 *Escherichia coli* isolates were recovered and genotyped by PCR. Among these, 101 strains (20.3%) were identified as enterotoxigenic *E. coli*, based on the presence of at least one fimbrial and one enterotoxin gene. The distribution of virulence factors among ETEC isolates was as follows: F5/K99F41 (50.5%), F5/K99 (39.6%), F41 (0.9%), and F6 (0.9%). The heat-stable enterotoxin gene STa was detected in all (100%) of the ETEC strains. The most prevalent virotypes were F5F41STa (50.5%) and F5STa (39.5%). Importantly, the genes encoding the fimbrial adhesins F4 and F18, as well as the enterotoxins LT and STb, were not detected in any of the analyzed isolates.

ETEC were detected in 17.2% (101/672) of the calves affected by neonatal calf diarrhea. Among the other pathogens investigated, BRV was the most frequently detected agent (22.2%), followed by BCoV (20.2%), and *Cryptosporidium* spp. (32.3%).

Mixed infections were observed in 26.3% of the calves. The most common co-infection was BRV + *Cryptosporidium* spp. (10.2%), followed by BRV + BCoV (8.5%) and BRV + ETEC (3.8%) (Table 3).

## 4. Discussion

This study investigates the prevalence of four major infectious agents associated with neonatal calf diarrhea in Northern Italy during 2020–2022. These four pathogens—BRV, BCoV, ETEC, and *Cryptosporidium parvum*—were selected based on their well-documented role as the principal etiological agents of NCD in the early postnatal period, particularly in calves up to 10 days of age. This selection aligns with previous studies, including the work by Wang et al. [29], which identified these agents as predominant in this age group. Furthermore, these pathogens are routinely included in the diagnostic panel of our laboratory for investigating enteric disorders in young calves. Focusing on these four agents allowed us to maintain methodological consistency and enhance the robustness and interpretability of our findings. Nonetheless, additional enteropathogens such as bovine norovirus, nebovirus, *Salmonella* spp., and *Clostridium perfringens* have also been associated with NCD [29] and may be considered in future studies to provide a more comprehensive understanding of the disease’s etiology. Environmental and management factors are known to influence the occurrence of NCD, including geography, climate, and breed susceptibility. However, the present research focuses specifically on a defined calf population from the Emilia-Romagna region, which is characterized by its flat topography and uniform farm distribution. Notably, NCD outbreaks were reported consistently throughout the year, with no clear correlation to seasonal changes, herd location, or breed. This observation highlights the need to further investigate non-climatic risk factors and underscores the importance of targeted preventive strategies.

NCD continues to be one of the most significant health concerns in young dairy calves. Effective control measures rely on early detection of clinical signs, accurate diagnosis, and an understanding of the predominant etiological agents within a specific region. In this context, distinguishing pathogenic strains of *Escherichia coli* from commensal strains is fundamental. In our study, ETEC was detected in 17.2% of diarrheic calves, a prevalence that is higher than that reported in several European countries such as the Netherlands (3%) and Norway (3%), but lower than values from Belgium (30–41%) (Table 4). The variation in prevalence rates highlights regional differences and underscores the need for local surveillance when designing preventive and therapeutic measures.

Molecular characterization revealed that the most common ETEC virulence gene detected in this study was the fimbrial adhesin F5, followed by F41. These findings align with previous reports from European countries, as summarized in the systematic review and meta-analysis by Kolenda et al. [11]. A recent study from Germany also reported a significant association between the presence of fimbrial adhesins F5, F17, and F41 and enteric disease in calves [30]. Although F17 was not included in the molecular panel applied in the present study—reflecting our routine veterinary diagnostic workflows, which typically prioritize fimbriae most consistently associated with classical ETEC pathotypes (i.e., F5 and F41) [11]—its involvement in pathogenic isolates reported elsewhere suggests that it may contribute to *E. coli* virulence; however, it is also found in ETEC strains isolated from healthy calves, so that warrants consideration in future investigations. 

ETEC F6 was also detected in this study, but its role in the etiology of calf diarrhea has to be better explained [11]. Kolenda et al. highlighted the need for further investigation regarding the role of ETEC F6 in neonatal calf diarrhea. Currently, there is limited data in the literature specifically addressing the prevalence of ETEC F6 in diarrheic and healthy calves. To our knowledge, no conclusive studies have definitively established the frequency or clinical relevance of ETEC F6 strains in healthy calves, which underscores the need for additional research in this area.

With regard to enterotoxins, STa was detected in 100% of ETEC isolates in our cohort. LT and STb enterotoxins were not detected, in agreement with prior studies [11,30,31]. These data contribute to the regional understanding of ETEC virulence profiles and support the design of effective vaccines—commercial or autogenous—tailored to the local pathogen landscape.

When considered alongside Table 4, our BRV prevalence of 22.2% is comparable to Belgium (26%) and higher than Norway (10%), but markedly lower than the 59% reported in Switzerland. Similarly, the BCoV prevalence of 20.2% observed in our cohort is notably higher than in the Netherlands (3%) and Norway (0%), yet lower than U.S. reports exceeding 30%. *Cryptosporidium* spp. prevalence in our study (32.3%) was intermediate, falling between the higher values seen in Switzerland (55%) and Wallonia (46%) and the lower values reported in Norway (4%). Among viral agents, BRV and BCoV were detected in 22.2% and 20.2% of Italian samples, respectively (Table 4). BRV is one of the most prevalent global causes of calf diarrhea, with detection rates ranging from 10% to over 60% depending on the region [31,32,33,34]. Our results are in line with the lower range of these values and are particularly comparable to data from Belgium (13–26%) and the Netherlands (18%), but markedly lower than those reported in Switzerland (59%) or Northern Ireland (32%). This variability likely reflects differences in age ranges considered, as our study included only calves ≤ 10 days of age, whereas most surveys included animals up to 21–30 days, a factor that may have contributed to the lower prevalence observed in our cohort. In contrast, the prevalence of BCoV in our study (20.2%) was higher than that reported in other European countries (ranging from 0% to 10%), highlighting the importance of local surveillance to capture regional epidemiological patterns. The BCoV prevalence observed in our study was higher than that reported in several European countries, such as Belgium (10%) and the Netherlands (3%) [13], though lower than findings from the U.S. (31.7%) [35]. In our study, we included only calves up to 10 days of age, whereas many other investigations considered calves up to 30 days old. This difference in age range may significantly contribute to the observed differences in prevalence. In addition to this, other well-known factors influencing the occurrence and distribution of NCD—such as farm management practices, biosecurity measures, herd density, and trading patterns—may also play a role. Such differences directly affect the detection and prevalence of etiological agents involved in neonatal calf diarrhea, complicating reliable comparative evaluations. These variations may be attributed to differences in testing protocols, herd demographics, and regional epidemiological dynamics (Table 4). *Cryptosporidium* spp. was identified in 32.3% of diarrheic calves, confirming its prominent role in calf enteritis. The persistence of *Cryptosporidium* spp. in the environment and its low infectious dose contribute to its widespread distribution [20]. Several studies conducted in countries such as Spain [36] and China [30] have reported different prevalence rates of *Cryptosporidium* spp. in calves. For example, Wang et al. (2023) [29] reported a prevalence of 35.4% in diarrheic calves aged ≤ 30 days in a study performed in China. Similarly, studies from Spain including only diarrheic calves up to 30 days of age reported prevalence rates ranging between 29% and 37% [36,37]. In contrast, other investigations often considered animals with a broader age range and, in some cases, included clinically healthy calves, which may partly explain the observed differences in prevalence. These differences in study design—particularly the inclusion of older and non-diarrheic animals—limit the comparability of results and likely account for the variation in prevalence observed across studies.

Among the mixed infections detected, the most frequently observed combinations were BRV and *Cryptosporidium* spp. (10.2%), followed by BRV and BCoV (8.5%), and BRV and ETEC (3.8%). The overall prevalence of mixed infections in our study was 26.3%, which is comparable to the range reported in previous literature, 20–35% in diarrheic calves [38,39]. Although these values do not indicate a high prevalence overall, the repeated occurrence of BRV in co-infections may suggest its frequent association with other pathogens. These interactions may prolong susceptibility and exacerbate clinical signs, as described by de la Fuente et al. [38] and Brunauer et al. [39]. Our findings are consistent with global trends, where BRV–*Cryptosporidium* spp. co-infection shows the highest pooled prevalence, followed by BRV-BCoV and BRV-ETEC [40]. BRV is also involved in the second and third most frequent co-infection combinations, even when it is not the predominant pathogen. This consistent presence in mixed infections may suggest a potential synergistic role of BRV in the pathogenesis of NCD or an increased susceptibility to co-infections in the presence of BRV. However, further investigations are necessary to better understand these interactions and their clinical significance.

Caution is warranted when comparing prevalence data across studies (Table 4). One of the main limitations of this study is the difficulty in comparing our findings with other published works, as these often differ in study design, the age range of calves included, and diagnostic methods employed. Such differences directly affect the detection and prevalence of etiological agents involved in neonatal calf diarrhea, complicating reliable comparative evaluations. Our results must also be interpreted considering the specific characteristics of our study design, which is based on passive surveillance: samples were collected from deceased calves voluntarily submitted by herd veterinarians following clinical outbreaks or unexplained increases in mortality. This sampling approach may have influenced the prevalence of certain pathogens compared to studies based on fecal sampling from live animals, as the health status at the time of sampling could bias detection rates. Furthermore, variables such as age group selection, diagnostic methods, sample types, and herd management practices—including herd size, biosecurity measures, animal husbandry systems, and trading patterns—can all significantly influence reported outcomes. Variables such as herd size, biosecurity measures, animal husbandry systems, and trading patterns all contribute to regional disparities in infection rates [30,40]. A comprehensive approach to farm biosecurity, alongside informed diagnostic strategies, remains key to mitigating NCD risks and improving calf health outcomes.

## 5. Conclusions

This study provides valuable epidemiological insights into the four major neonatal calf diarrhea pathogens involved in NCD outbreaks in the Emilia-Romagna region of Northern Italy. Our findings confirm that BRV, *Cryptosporidium* spp., BCoV, and ETEC are key contributors to NCD in calves up to 10 days old. Notably, mixed infections were frequent, with the most common co-infections involving BRV and *Cryptosporidium* spp., followed by BRV with BCoV and ETEC, suggesting potential synergistic interactions that may exacerbate disease severity.

The relatively low prevalence of BRV compared to other European studies is likely related to the narrower age range of sampled calves, emphasizing the importance of standardized age criteria in epidemiological studies. Moreover, the detection of ETEC strains with virulence factors less commonly studied, such as F6 adhesins, highlights the need for further investigation into their role in NCD pathogenesis.

Our results underscore the necessity of routine, multi-agent diagnostic protocols for accurately identifying the complex etiology of NCD and informing effective control strategies. This passive surveillance study also lays the groundwork for future active surveillance programs in high-density livestock areas, aiming to better prevent and manage neonatal calf diarrhea outbreaks.

## Figures and Tables

**Table 1:** Anatomopathological lesions observed in the calves included in this study at necropsy. Lesions are expressed as a percentage of the total number of calves examined.

Lesion Category	Lesion Subtype	Percentage (%)
Abomasitis	Absent	12
	Catarrhal	83
	Abomasal ulceration	5
Enteritis	Absent	0
	Catarrhal	89
	Catarrhal–hemorrhagic	8
	Fibrinous	3
Lymphadenomegaly	Absent	22
	Mesenteric	74
	Generalized	4

**Table 2:** List of primers and probes used in multiplex RT-PCR for BRV and BCoV.

Primer/Probe Name	Sequence 5′-3′	Target	Reference
BRV-F5	TCATTTCAGTTGATGAGACCACC	BRV A (VP6)	[26]
BRV-F6	ATTCAATTCTAAGCGTGAGTCTAC		
BRV-Probe	TexasRed-AATATGACACCAGCGGTAGCGGC-(TexasRed-BHQ2)		
BCoV-fwd	CTAGTAACCAGGCTGATGTCAATACC	BCoV (N)	[27]
BCoV-rev	GGCGGAAACCTAGTCGGAATA		
BCoV-Probe	CGGCTGACATTCTCGATC (FAM-MGBNFQ)		

**Table 3:** Frequency and combinations of mixed infections among diarrheic calves. *BRV*, bovine rotavirus; *BCoV*, bovine coronavirus; *ETEC*, enterotoxigenic *Escherichia coli*; *Cryptosporidium* spp. Values are reported as % (n).

Mixed Infection	Frequency (%)
BRV–*Cryptosporidium* spp.	10.2
BRV–BCoV	8.5
BRV–ETEC	3.8
BRV–BCoV–*Cryptosporidium* spp.	2.4
BCoV–*Cryptosporidium* spp.	1.2
BCoV–ETEC	0.1
ETEC–*Cryptosporidium* spp.	0.1

**Table 4:** The prevalence estimate of major neonatal calf diarrhea pathogens in European calves (adapted from [15]). * Current study; -, data not available.

Etiological Agent	Belgium	Wallonia (Belgium)	Netherlands	Northern Ireland	Norway	Switzerland	Italy *
CALVES’ AGE RANGE (days)	-	0–30	1–21	-	1–30	1–20	1–10
ETEC	41%	30%	3%	7%	3%	6%	17.20%
BRV	13%	26%	18%	32%	10%	59%	22.20%
BCoV	-	10%	3%	4%	0%	6%	20.20%
*Cryptosporidium* spp.	23%	46%	28%	35%	4%	55%	32.30%

## Data Availability

Data are contained within the article.
